# First *in vivo* robotic-assisted pulsed field ablation for atrial fibrillation in a swine model: a case report

**DOI:** 10.1093/ehjcr/ytaf518

**Published:** 2025-11-18

**Authors:** Nianqin Zhang, Chi Cai, Yan Yao, Ligang Ding, Wei Hua

**Affiliations:** State Key Laboratory of Cardiovascular Disease, Fuwai Hospital, National Center for Cardiovascular Diseases, Chinese Academy of Medical Sciences and Peking Union Medical College, No. 167 Beilishi Rd, Xicheng District, Beijing 100037, People’s Republic of China; State Key Laboratory of Cardiovascular Disease, Fuwai Hospital, National Center for Cardiovascular Diseases, Chinese Academy of Medical Sciences and Peking Union Medical College, No. 167 Beilishi Rd, Xicheng District, Beijing 100037, People’s Republic of China; State Key Laboratory of Cardiovascular Disease, Fuwai Hospital, National Center for Cardiovascular Diseases, Chinese Academy of Medical Sciences and Peking Union Medical College, No. 167 Beilishi Rd, Xicheng District, Beijing 100037, People’s Republic of China; State Key Laboratory of Cardiovascular Disease, Fuwai Hospital, National Center for Cardiovascular Diseases, Chinese Academy of Medical Sciences and Peking Union Medical College, No. 167 Beilishi Rd, Xicheng District, Beijing 100037, People’s Republic of China; State Key Laboratory of Cardiovascular Disease, Fuwai Hospital, National Center for Cardiovascular Diseases, Chinese Academy of Medical Sciences and Peking Union Medical College, No. 167 Beilishi Rd, Xicheng District, Beijing 100037, People’s Republic of China

**Keywords:** Pulsed field ablation, Robotic-assisted procedure, Swine model, Case report

## Abstract

**Background:**

Robotic-assisted procedures are rapidly advancing in interventional cardiology and have shown advantages in performing atrial fibrillation ablation. However, their application in combination with pulsed field ablation (PFA) remains unexplored.

**Case summary:**

We report the first *in vivo* experience of robotic-assisted PFA. The robotic-assisted PFA system comprises of a robotic articulating arm with three sterile, single-use functional modules, a workstation console, and a control computer. Following transseptal puncture and electrical mapping, two experienced operators performed pulmonary vein isolation and superior vena cava isolation in a swine model using the robotic system integrated with a guiding sheath and a PFA catheter. The procedure was conducted under fluoroscopy, while operators controlled the system remotely via the console in a zero-radiation environment. Post-procedural voltage mapping and histological analysis confirmed the feasibility of robotic-assisted PFA.

**Discussion:**

The primary advantage of robotic-assisted PFA is the potential to enable remote operation. It may also improve catheter stability and reduce the learning curve for performing PFA. Potential risks of robotic-assisted PFA include violent damage to cardiac tissue and complications associated with PFA. The robotic-assisted PFA system is sensitive and accurate when operated on a swine model, though several challenges exist in translating findings from a swine model to human clinical practice, and further studies are needed to evaluate its safety and efficacy before clinical implementation.

Learning points

- The success of this first *in vivo* robotic-assisted pulsed field ablation (PFA) study demonstrates the potential of robotic-assisted PFA for clinical application.
- The major advantage of robotic-assisted PFA is the ability to facilitate remote operation, including in a zero-radiation environment, while a potential risk of the robotic-assisted PFA is the violent damage to cardiac tissue.

## Introduction

Robotic-assisted procedures have emerged as a promising trend in interventional therapy, offering the advantages of remote operation and enhanced procedural stability. In atrial fibrillation (AF) ablation, significant advancements have been made in robotic-assisted radiofrequency ablation, with systems such as the Hansen Sensei robotic system and remote magnetic navigation systems.^1,2^ Pulsed field ablation (PFA) is a novel, non-thermal technique for treating AF, with efficacy and safety comparable to conventional radiofrequency or cryoballoon ablation, as demonstrated in large randomized trials.^3^ However, the integration of robotics with PFA has not been previously explored. This report presents the first *in vivo* experience of robotic-assisted PFA in a swine model, demonstrating the feasibility of combining robotic control with a multielectrode PFA catheter.

## Summary figure

**Figure:**
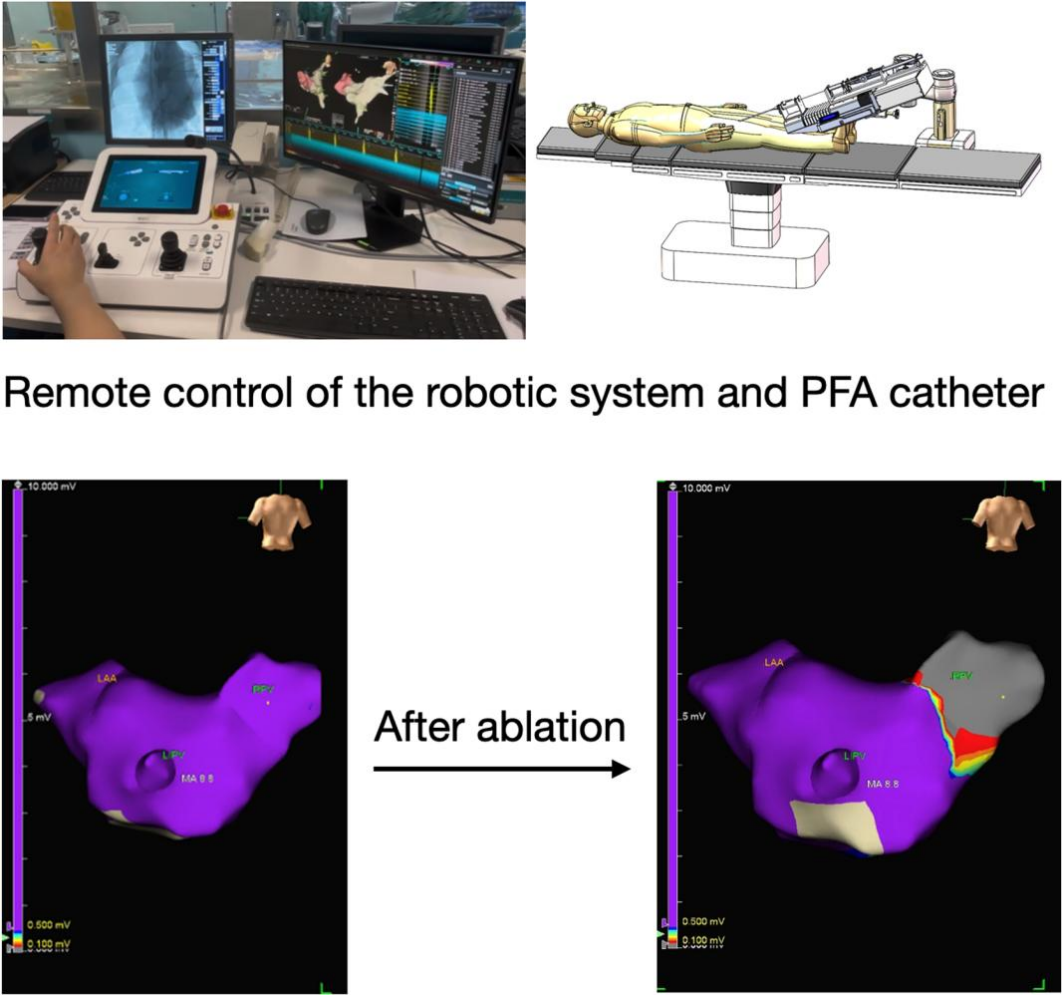
The first in-vivo robotic-assisted pulsed field ablation was performed on a swine model. The control of the robotic arm was achieved remotely via the workstation console. Post-procedural mapping of the left atrium shows successful pulmonary vein isolation. LAA, left atrial appendage; LIPV, left inferior pulmonary vein; RPV, right pulmonary vein.

## Case presentation

The robotic-assisted PFA system used in this study comprises of a robotic articulating arm with three sterile, single-use functional modules, a workstation console with joysticks and buttons, and a control computer. This platform is adapted from the transcatheter edge-to-edge repair robotic system described by Zhu *et al.*^4^ The joysticks and buttons on the console control the robotic arm and functional modules to adjust the position and angle of the guiding sheath and the PFA catheter inside it, including deployment and compression of the ablation loop. The control computer serves as the central processing unit, transmitting signals and providing feedback to the operator. The robotic-assisted PFA system underwent extensive preclinical validation prior to its *in vivo* use, including bench testing of catheter manoeuvrability in simulated cardiac anatomies, confirming its ability to maintain stable contact and navigate precisely. Based on these results, we performed the first *in vivo* study. The study protocol involved performing pulmonary vein isolation (PVI) and superior vena cava (SVC) isolation on two swine by two experienced operators. Before the procedure, operators were trained with the manual and performed hands-on practice for 30 min.

Two female swine (weight 58 ± 1 kg, age 4 months) were obtained from an approved vendor. All animals received humane care in compliance with the National Institutes of Health Guide for the Care and Use of Laboratory Animals (revised 2011), and the study protocol was approved by a local Animal Ethics and Welfare Committee. All procedures were performed under general anaesthesia and mechanical ventilation. The animals were sedated with an intramuscular injection of tiletamine hydrochloride and zolazepam hydrochloride (2 mg/kg), along with xylazine hydrochloride (2 mg/kg), and anaesthesia was maintained with 3% sevoflurane.

**Figure 1:**
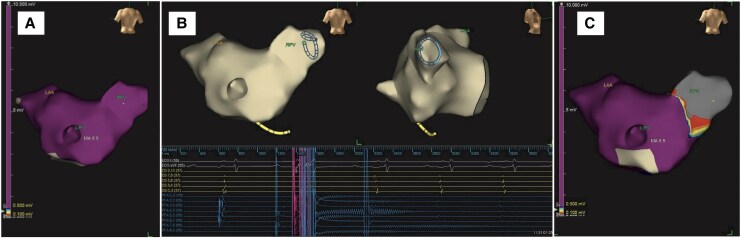
Right pulmonary vein voltage before (*A*), during (*B*), and after (*C*) pulsed field ablation. Panel (*B*) shows the immediate disappearance of pulmonary vein potentials when the robotic-assisted pulsed field ablation system delivers electrical pulses. LAA, left atrial appendage; LIPV, left inferior pulmonary vein; RPV, right pulmonary vein.

**Figure 2:**
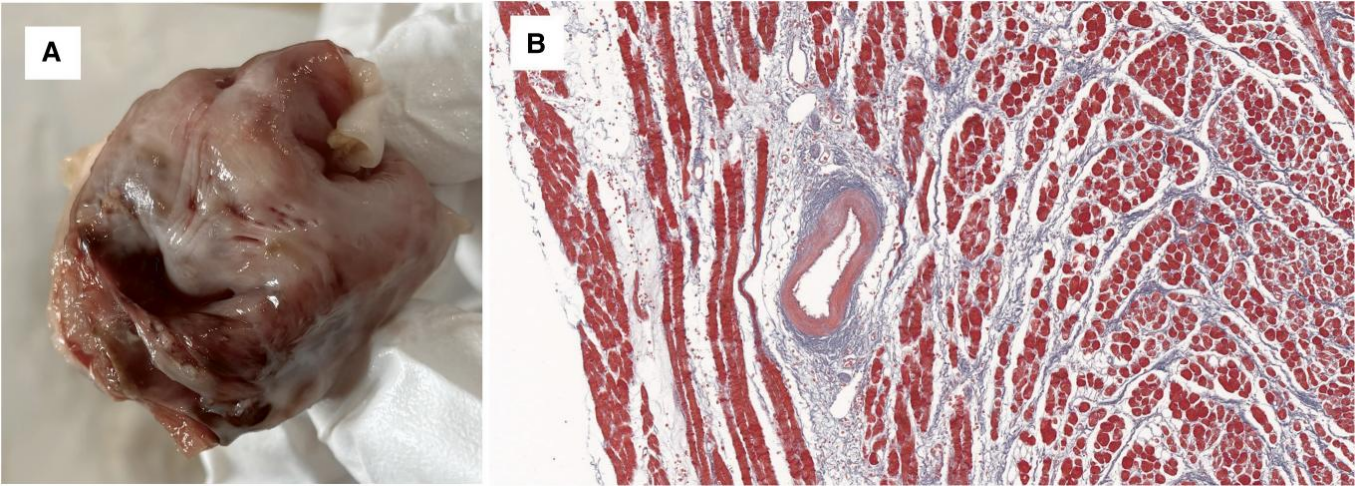
Pulsed field ablation lesion. (*A*) Anatomical examination with triphenyltetrazolium chloride staining, with the lesion appearing white. (*B*) Histological analysis with Masson’s trichrome staining showing that pulsed field ablation spares arteries.

Transseptal puncture and electrical mapping were performed manually, with mapping conducted using the EnSite X system (Abbott, Inc.). Following the setup of the robotic system, a circular multielectrode array PFA catheter (ShengDaJi Medical) and guiding sheath (ShengDaJi Medical) were mounted on the robotic arm and paired with the functional modules. The operators then moved to a zero-radiation environment to operate the robotic system remotely. The correct positioning of the guiding sheath and PFA catheter within the left atrium was confirmed via fluoroscopy. Subsequently, the ablation loop was manoeuvred to make contact with the right common pulmonary vein. A single PFA application was defined as four biphasic, bipolar pulse trains, each lasting 200 ms at 1500 V. Voltage amplitude changes were monitored and recorded. The ablation loop was then rotated 90° after each application to complete PVI. The ablation loop was then navigated to the left superior pulmonary vein, the left inferior pulmonary vein, and the SVC to complete the procedure. Post-procedural mapping confirmed successful PVI and SVC isolation (*Figure 1*). Operator feedback was collected using a five-point scale: ‘poor’ (lowest), ‘fair’, ‘acceptable’, ‘good’, and ‘excellent’ (highest). For sensitivity, both operators described it as ‘good’. Accuracy was considered ‘acceptable’ by one operator and ‘good’ by the other. Manoeuvrability was regarded as ‘acceptable’ by one operator and ‘good’ by the other. Post-procedural anatomical and histological analysis (TTC and Masson's trichrome staining) demonstrated well-demarcated transmural lesions with preserved arterial structures (*Figure 2*).

## Discussion

In this study, we present the first *in vivo* experience of robotic-assisted PFA. Operators successfully performed PVI and SVC isolation using remote console control in a zero-radiation environment. The navigation of the guidewire, guiding sheath, and PFA catheter was accurate under fluoroscopic guidance, and post-procedural mapping and histology confirmed lesion effectiveness. Overall, we found the robotic system to be sensitive and manoeuvrable, though it requires practice for effective manipulation. Currently, it is only compatible with the specific PFA catheter used in this procedure; future models aim to accommodate a broader range of PFA catheters.

A major advantage of robotic-assisted PFA is its ability to facilitate remote operation, which has the potential to significantly benefit patients, especially if future integration with mapping and transseptal puncture is achieved. Additionally, it may improve catheter stability and reduce the learning curve for performing PFA. Prior studies have shown that robotic-assisted radiofrequency can create more permanent lesions compared to manual procedures, likely due to improved catheter stability.^5^ We expect similar benefits from robotic-assisted PFA. However, robotic-assisted PFA also carries risk. A potential risk of the robotic-assisted PFA is the violent damage to cardiac tissue. In our study, despite console manipulation, the catheter occasionally appeared stationary under fluoroscopy because it became trapped in myocardial tissue. Integration of a real-time contact force feedback, which is not yet included in the current robotic system setup, could help prevent excessive force application and unintended tissue damage. Other potential risks include cardiac tamponade, excessive myocardial damage, and extracardiac injury, consistent with known PFA complications.^3,6^ Given that PFA requires less procedural time, is easier to perform, and has a higher first-pass isolation rate than radiofrequency or cryoballoon ablation, and with comparable complications,^7^ robotic-assisted PFA could be safer and more efficient than robotic-assisted radiofrequency or cryoballoon ablation. However, further studies are needed to compare robotic-assisted ablation with different energies.

Several challenges exist in translating findings from a swine model to human clinical practice. First, anatomical differences between swine and human hearts—especially in pulmonary vein morphology—may affect catheter navigation and lesion formation. Second, although the robotic-assisted PFA system demonstrated feasibility in this acute setting, long-term safety remains unassessed. Third, our study included only two swine, so results may differ in larger studies or in human populations. Finally, regulatory approval and operator training will be needed before clinical use.

## Conclusion

This is the first reported *in vivo* study demonstrating the feasibility of robotic-assisted PFA. Further studies are needed to evaluate its long-term safety and efficacy before clinical implementation.

## Lead author biography


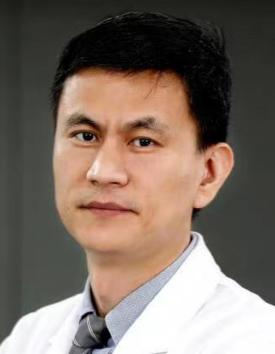


As a chief physician, his current clinical practice is centred on complex catheter ablation procedures. His research primarily concentrates on the translational and clinical investigation of pulsed field ablation for the treatment of atrial and ventricular arrhythmias.

**Funding:** This study was supported by grants from the Noncommunicable Chronic Diseases-National Science and Technology Major Project (2024ZD0521900), and by the Clinical and Translational Medicine Research Program (grant number 2023-LC05) of the Chinese Academy of Medical Sciences.

## Data Availability

The data that support the findings of this study are available from the corresponding author.
